# The Genomics of Neonatal Abstinence Syndrome

**DOI:** 10.3389/fped.2017.00176

**Published:** 2017-08-22

**Authors:** F. Sessions Cole, Daniel J. Wegner, Jonathan M. Davis

**Affiliations:** ^1^The Edward Mallinckrodt Department of Pediatrics, Division of Newborn Medicine, Washington University School of Medicine, St. Louis Children’s Hospital, St. Louis, MO, United States; ^2^The Department of Pediatrics, Division of Newborn Medicine, The Floating Hospital for Children at Tufts Medical Center, The Clinical and Translational Science Institute, Tufts University, Boston, MA, United States

**Keywords:** genomics, opioids, neonatal abstinence syndrome, precision medicine, treatment

## Abstract

Significant variability has been observed in the development and severity of neonatal abstinence syndrome (NAS) among neonates exposed to prenatal opioids. Since maternal opioid dose does not appear to correlate directly with neonatal outcome, maternal, placental, and fetal genomic variants may play important roles in NAS. Previous studies in small cohorts have demonstrated associations of variants in maternal and infant genes that encode the μ-opioid receptor (*OPRM1*), catechol-*O*-methyltransferase (*COMT*), and prepronociceptin (*PNOC*) with a shorter length of hospital stay and less need for treatment in neonates exposed to opioids *in utero*. Consistently falling genomic sequencing costs and computational approaches to predict variant function will permit unbiased discovery of genomic variants and gene pathways associated with differences in maternal and fetal opioid pharmacokinetics and pharmacodynamics and with placental opioid transport and metabolism. Discovery of pathogenic variants should permit better delineation of the risk of developing more severe forms of NAS. This review provides a summary of the current role of genomic factors in the development of NAS and suggests strategies for further genomic discovery.

## Introduction

An epidemic of neonatal abstinence syndrome (NAS) currently exists due to dramatic increases in prenatal opioid exposure. Significant variability has been noted in the incidence and severity of NAS among neonates exposed to prenatal opioids. The promise of precision medicine has prompted increasing interest in discovery of genomic variants that could better predict the development and severity of NAS and suggest new therapeutic approaches for both prevention and treatment (1–3). Although the prevalence of NAS has remained stable between 1997 and 2011 in England and Australia (4), NAS has increased significantly in the United States over the past several years from 7 cases/1,000 neonatal intensive care unit admissions in 2004 to 27 cases/1,000 admissions in 2013 (5). The economic (now >$1.5 billion per year in the United States), emotional, and social burdens associated with NAS are substantial due to the frequent need for prolonged hospitalizations and the increased risk of adverse long-term neonatal neurodevelopmental outcomes (6, 7). While most neonates developing NAS respond well to treatment with opioids, a subset of exposed infants will require additional pharmacological therapy (e.g., phenobarbital, clonidine) and longer hospitalizations (1, 8–10). Multiple environmental factors such as maternal socioeconomic status and prenatal exposure to other illicit and prescription drugs contribute to neonatal outcome. Precision medicine initiatives involving genomic-based prediction of NAS severity are particularly attractive due to previous associations between individual genomic variants and outcome, poor predictive accuracy of most clinical and demographic factors, and rapidly falling sequencing costs (2, 11, 12). Epigenetic regulation of genes associated with maternal and fetal drug metabolism and placental drug metabolism and transport may also contribute to disease severity and have been reviewed previously (13).

## Associations of Genomic Variants with NAS Risk and Severity

Based on twin studies, opioid addiction is heritable (14). This heritability has prompted case–control studies that have examined the association of several single-nucleotide polymorphisms (SNPs) in genes known to be involved in opioid metabolism and addiction with length of hospitalization, need for pharmacologic treatment, and total days of opioid treatment in neonates with NAS (12) (Table 1). Results suggest that SNPs in *both* mother and infant are associated with severity of NAS (2, 15–17). For example, in studies of opioid-exposed newborn infants and their mothers (2, 15), SNPs in maternal and infant genes that encode the μ-opioid receptor (*OPRM1*), catechol-*O*-methyltransferase (*COMT*), and prepronociceptin (*PNOC*) were associated with differences in neonatal outcomes (length of hospitalization and reduced need for treatment with two medications). While the SNPs chosen for study are known to have important physiologic roles in opioid drug response and withdrawal in adults, the effect sizes of these SNPs have been modest and have not had a significant impact on clinical practice (2, 16). In addition, differences in allele frequencies suggest that the use of a limited number of SNPs as biomarkers of disease severity will require race-/ethnicity-based stratification and assessment of admixture (Table 1). Functional annotation and prediction programs may permit improved risk evaluation of individual SNPs (18) and comparison of the contributions of maternal and infant drug metabolism to risk of NAS. Finally, the complexity of the genomic architecture, developmental regulation of gene expression, and the contributions of maternal medications and confounding diagnoses will require further refinement of SNP-based strategies before they can be used clinically.

**Table 1:** Genes associated with NAS phenotype.

Gene	SNP	Genotype	Associated NAS phenotype	Annotation	CADD score^c^	Allele frequencies		
						EA	Afr	Latino
*OPRM1*	rs1799971	G allele^a^	Less likely to receive treatment; shortened length of stay	Missense Asn133Asp	24.1	0.13	0.03	0.21

*COMT*	rs4680	G allele^a^	Less likely to receive treatment with two or more medications; shortened length of stay	Missense Val158Met	11.4	0.48	0.69	0.6
	rs4680	G allele^b^	Less likely to receive treatment with two medications		11.4			
	rs740603	A allele^a^	Less likely to receive pharmacological treatment; shortened length of stay	Intronic	0.2	0.49	0.56	0.61
	rs740603	A allele^b^	Less likely to receive any pharmacological treatment		0.2			

*PNOC*	rs351776	A allele^a^	More likely to be treated with two medications; longer length of stay	Intronic	1.3	0.44	0.78	0.69
	rs351776	A allele^b^	More likely to receive treatment with two medications; longer length of stay		1.3			
	rs2614095	A allele^a^	More likely to be treated with two medications	Intronic	2.9	0.46	0.11	0.27
	rs4732636	A allele^a^	Less likely to receive medication treatment	Upstream	6.3	0.28	0.1	0.36

*OPRK1*	rs702764	C allele^a^	More likely to receive treatment with two medications	Synonymous	0.4	0.13	0.49	0.28

*OPRD1*	rs204076	A allele^b^	More likely to receive treatment with two medications; longer length of stay	Downstream	5.6	0.66	0.83	0.82

*DRD2*	rs1799732	Deletion	Less likely to require treatment with medication	Upstream	N/A	0.1	0.5	0.12

*^a^Infant allele*.

*^b^Maternal allele*.

*^c^Combined annotation-dependent depletion (17)*.

*EA, European–American; Afr, African; N/A, not applicable (frame shift)*.

## Future Directions for Genomics of NAS Research

Extension of current SNP-based studies of the genomics of NAS may benefit from leveraging available, diverse large exome and genome databases (e.g., http://gnomad.broadinstitute.org/) to evaluate the predicted function and frequency of both common and rare SNPs in genes known to be associated with heritable addiction or alterations in drug metabolism. Falling sequencing costs will permit complete genomic resolution for discovery of clinically actionable genomic variants in individual mother/infant dyads. This approach should avoid the need to rely on linkage disequilibrium or imputation. Unbiased whole exome or genome sequencing would permit the evaluation of the contributions of rare variants in both recognized and unrecognized genes to NAS severity. The statistical challenges associated with rare variant discovery may be partially addressed by using family study design (e.g., twin studies) to reduce the contributions of highly diverse genomic backgrounds to phenotype and multiple confounding co-variables present in case–control studies. Examining infants with the extremes of NAS severity may also improve statistical power to identify relevant genomic variants (19). A review of copy number and structural variant databases to identify individuals who are haploinsufficient for specific genes might also provide a strategy to evaluate functional impact of loss of function alleles (20). Standard phenotyping that incorporates predictions of functional impact on the metabolism of opioids, as has been proposed for the cytochrome P450 genes, along with currently accepted measures of NAS severity may also be useful (21, 22). Developmental regulation of relevant drug metabolizing and placental transport genes will likely require model systems for testing gestational age-specific effects of individual variants (23, 24). Finally, development of statistical approaches that incorporate confounding variables like breastfeeding, smoking, and use of protocol-driven medication weaning strategies (25) will be important to compare the contributions of genomic variants with these kinds of clinical variables. Application of precision medicine to the problem of NAS could benefit the many adults and neonates exposed to opioids by providing individualized risk prediction and leveraging discovery of candidate genes for development of novel therapeutic strategies (3, 12). For example:

(A) If we can establish high-risk genetic profiles in young women, can we then limit opioid exposure?
(B) Can we establish low-risk genetic profiles in young women and safely wean them off their medication-assisted treatment without an increased risk of relapse?
(C) If high-risk profiles are found in pregnant women, can we intervene and modify risk factors such as limiting cigarette smoking and polypharmacy?
(D) With high-risk profiles identified in neonates, could we administer low doses of opioids immediately after birth and limit the development and severity of NAS?
(E) If lower risk profiles are identified in neonates, could we send them home earlier and carefully follow them up as an outpatient?
(F) Can pharmacogenomics identify optimal treatment strategies (i.e., best practices) for the mother and her neonate?
(G) If genetic risk factors can be combined with clinical and demographic variables, can we develop “risk assessment” models that can be applied to neonates, older children, and adults to determine the risk of addiction following exposure to opioids (e.g., low, medium, high)?

These are some of the potential benefits of a more precision medicine-guided approach that could be adopted with rapid development of novel genomics methodologies. What is most important is sufficient funding, and legislative efforts are currently driving this innovation and offer a path forward to address NAS in this highly vulnerable population.

## Author Contributions

FC and JD conceptualized and drafted the entire manuscript; DW contributed data analysis.

## Conflict of Interest Statement

The authors declare that the research was conducted in the absence of any commercial or financial relationships that could be construed as a potential conflict of interest. The reviewer, TF, and handling editor declared their shared affiliation, and the handling editor states that the process nevertheless met the standards of a fair and objective review.
